# A coupled model for public health risk: hazard and urban vulnerability in 18 cities in Sichuan, China

**DOI:** 10.3389/fpubh.2025.1639263

**Published:** 2025-09-17

**Authors:** Lijuan Liu, Jian Qiu, Mian Yang, Suling Mao, Xiuwei Cheng, Ming Cui, Weile Li

**Affiliations:** ^1^School of Architecture, Southwest Jiaotong University, Chengdu, China; ^2^Southwest Jiaotong University Chengdu Design Institute, Chengdu, China; ^3^Research Center of Spatial Planning and Engineering Design of SWJTU, Chengdu, China; ^4^Faculty of Art, Sichuan Tourism University, Chengdu, China; ^5^Sichuan Center for Disease Control and Prevention, Chengdu, China; ^6^Chengdu Institute of Planning and Design, Chengdu, China; ^7^State Key Laboratory of Geohazard Prevention and Geoenvironment Protection, Chengdu University of Technology, Chengdu, China

**Keywords:** public health emergencies, risk coupling model, infectious disease hazards, urban vulnerability, entropy method, coupling coordination degree (CCD) model

## Abstract

**Introduction:**

In the research and practice of disaster prevention/mitigation and urban resilience development, although existing studies have conducted multidimensional assessments of urban vulnerability to hazards and infectious disease risks, limitations persist—such as the lack of bidirectional coupling mechanism analysis and a disconnection from planning implementation. These constraints hinder the systematic governance of public health risks and the advancement of resilient city development.

**Method:**

This study selects 18 prefecture-level cities in Sichuan Province as case studies. By employing the entropy method and coupling coordination degree (CCD) model, we construct a “hazard-vulnerability” risk coupling model to systematically analyze the coupling coordination mechanisms, identify key influencing factors, and propose optimization pathways.

**Results:**

(1) The coupling coordination degree (CCD) between infectious disease hazards and urban vulnerability in Sichuan Province remains at a relatively low level overall (mean = 0.384). Specifically, Chengdu demonstrates a “low vulnerability-high hazard” characteristic (0.031), while Guangyuan and Panzhihua exhibit optimal coordination states (0.655 and 0.649 respectively). (2) The region generally follows the distribution pattern where lower CCD corresponds to higher risk levels. The coordinated development types show dispersed spatial distribution, whereas recession-maladjusted types are predominantly concentrated in the Chengdu Plain and southern Sichuan regions. (3) Among CCD subtypes, the “hazard-deficit” type emerges as the dominant pattern. (4) Economic-spatial-social-environmental factors demonstrate not only significant interaction effects but also pronounced spatial heterogeneity characteristics.

**Conclusion:**

Based on spatial coupling theory, this study innovatively constructs a “hazard-vulnerability” risk coupling model, which expands traditional risk assessment and urban vulnerability evaluation theories, providing a novel research perspective for urban risk management and regional sustainable development. The research results offer important quantitative evidence for formulating regionally differentiated public health strategies.

## 1 Introduction

Public health emergencies, as global crises, have seen their impact mechanisms and spatial distribution characteristics become a cutting-edge research topic in interdisciplinary studies. From a historical perspective, from the Plague of Justinian to the COVID-19 pandemic, such events have not only caused significant casualties and economic losses (1, 2), but have also reshaped urban development trajectories through complex spatial interaction mechanisms (3). In contemporary urbanization processes, the concentration of factors and flow networks have simultaneously improved the efficiency of medical resource allocation while significantly increasing pathogen transmission risks (4). Therefore, it is essential to establish a scientific risk assessment framework to effectively implement urban planning strategies, public health policies, and emergency management measures.

In the field of infectious disease transmission mechanisms, significant academic progress has been achieved. Dai et al. systematically demonstrated the potential risks of respiratory disease transmission via aerosols in high-density urban environments (5), while Ruiz-Herrera et al. mathematically quantified the critical role of population mobility in epidemic spread (6). Notably, however, these studies primarily focus on the transmission dynamics of pathogens themselves, failing to adequately account for the regulatory effects of urban complex systems on transmission processes.

Meanwhile, urban vulnerability studies have thoroughly examined the impact of socioeconomic factors on public health emergency response capabilities (7), explicitly identifying spatial elements as key variables influencing disease transmission. These studies reveal how urban–rural spatial organization patterns critically determine epidemic prevention efficacy (8). Particularly noteworthy is Rahayu et al.’s research demonstrating how disparities in urbanization levels and regional development imbalances exacerbate health vulnerabilities, leading to spatial mismatches between public health service provision and disease burdens (9). However, these studies generally overlook pathogen-specific transmission routes and pathogenic mechanisms. Such disciplinary fragmentation has resulted in significant theoretical limitations and practical blind spots in existing risk assessment frameworks.

Existing studies have also revealed that the impact of urbanization on infectious disease transmission exhibits significant regional heterogeneity (10). This spatial variation manifests not only in the geographical disparities of transmission risks, but also triggers multi-level cascading effects within urban systems through the shockwaves of public health emergencies. Specifically, public health crises have both intensified the polarization of pre-existing patterns in disease transmission and socioeconomic spatial differentiation (11), while simultaneously giving rise to new vulnerability dimensions such as disparities in spatial accessibility, environmental justice imbalances, and inequitable health resource allocation (12). The spatial coupling and synergistic effects of these multidimensional inequities not only exacerbate the degree of risk heterogeneity in urban systems, but also pose systemic challenges to conventional public health risk management paradigms.

Building upon these research findings, scholars have begun to re-examine the adaptability of traditional urbanization models and advocate for establishing systematic, multi-tiered, and dynamically evolving urban resilience frameworks (13). Grounded in urban political ecology theory, Gandy developed the “Zoonotic City” analytical framework, emphasizing that urbanization processes must be integrated with epidemiological characteristics to fully capture the complex interactions between health threats and environmental changes (14). Furthermore, Yang et al.’s empirical study in Hubei Province proposed that post-pandemic urban development should transcend mere economic agglomeration and scale expansion, shifting toward a new model prioritizing public service enhancement and amenity optimization (15). Additionally, Pacheco et al.’s systematic review demonstrated that increasing accessible public spaces and optimizing their adaptive use during health crises are emerging as critical innovations in urban design (16). These research advances provide vital theoretical foundations and practical pathways for constructing more resilient urban systems.

Through an in-depth analysis of current research advancements, three critical theoretical gaps remain to be addressed in the study of interactions between infectious diseases and urban systems. First, existing research paradigms are predominantly limited to unidirectional linear analyses, focusing either on the mechanisms of disease transmission and the impact of epidemics on urban systems, or examining the influence of urban factors on disease spread in isolation (17). This fragmented research perspective has led to insufficient understanding of the complex interaction mechanisms between hazards and vulnerabilities. Second, at the methodological level, current risk assessment frameworks lack adequate capacity to analyze the formation mechanisms of micro-scale risk heterogeneity within cities, making it difficult to effectively identify key drivers of risk differentiation across different regions (18). More crucially, despite substantial evidence demonstrating significant correlations between spatial organization patterns and epidemic control effectiveness, there remains a lack of integrated frameworks to effectively translate risk assessment results into urban planning intervention measures (19). These theoretical and methodological limitations urgently call for establishing systematic, multidimensional, and dynamic infectious disease risk assessment systems, and implementing precise interventions through scientific risk management approaches (20).

In summary, this study systematically conducted public health emergency risk assessment research using 18 prefecture-level cities in Sichuan Province as case studies. Methodologically, we first constructed comprehensive evaluation index systems for both hazard and urban vulnerability, employing the entropy method to determine indicator weights, subsequently measuring their index levels and analyzing spatial distribution characteristics. Building upon this foundation, the integrated risk assessment model quantified disaster risk levels and generated risk maps, verifying the effectiveness of the index system as an informative indicator for actual cumulative infection data (as the level of risk alone can be an informative indicator for all such issues). Furthermore, the CCD model was applied to analyze the spatial coupling relationship between hazards and vulnerability, not only classifying coupling coordination types but also identifying key risk drivers for each category. Ultimately, empirical analysis based on pandemic infection growth data validated the reliability of the coupled risk assessment results. By developing the “hazard-vulnerability” risk coupling model, this study expands traditional risk assessment theory and provides scientific support for formulating effective risk management measures and urban planning strategies (21). The research holds significant theoretical and practical value for integrated disaster prevention and mitigation system planning, resilient city construction, and sustainable development.

## 2 Materials and methods

### 2.1 Study area and data sources

#### 2.1.1 Study area

Sichuan Province, located in southwestern China, plays a pivotal role in major national strategies such as the Western Development Program, poverty alleviation initiatives, and the Chengdu-Chongqing Economic Circle development. Despite its well-developed transportation network that facilitates efficient population mobility and material flows, the urban system remains incomplete. Expect Chengdu, the province lacks other megacities and Type I large cities, and has only three Type II large cities, resulting in population shrinkage among small-medium cities and excessive con-centration in central urban areas. Although Sichuan ranks fifth nationally in regional GDP, its economic development shows significant spatial disparities. The overall development level remains relatively lagging, with pronounced urban–rural gaps, uneven resource allocation, and low spatial safety resilience. Historically prone to earth-quakes and epidemics, the province’s health risks have been further exposed during recent major pandemic outbreaks.

**Figure 1:**
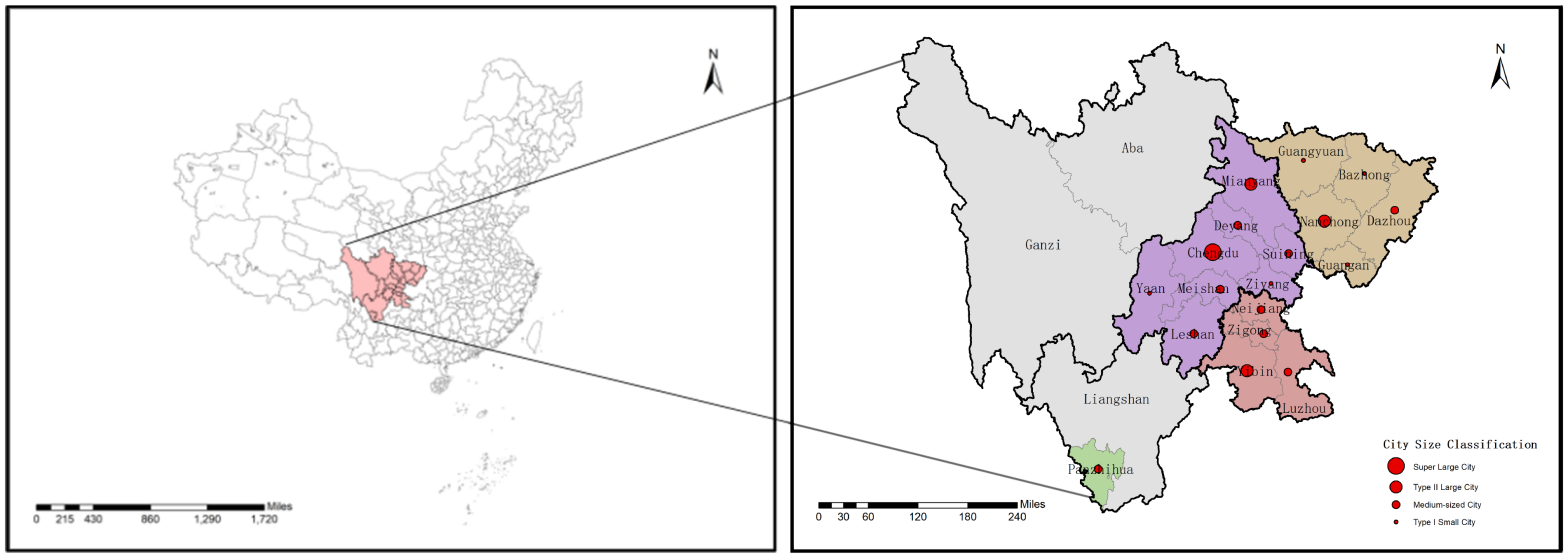
The location of Sichuan Province.

This study examines 18 prefecture-level and higher cities within four major economic zones of Sichuan (Table 1; Figure 1). The provincial capital Chengdu, with an urban population of 13.34 million, ranks as China’s 6th megacity. Its rapid economic development has created significant population siphon effects, with its massive urban population far exceeding other cities in the province. The province’s urban system comprises three Type II large cities (Mianyang, Nanchong, and Yibin), nine medium-sized cities (Luzhou, Dazhou, Zigong, Suining, Leshan, Meishan, Panzhihua, Deyang, and Neijiang), and five Type I small cities (Guangyuan, Bazhong, Ziyang, Guang’an, and Ya’an), collectively constituting a hierarchical urban network beneath Chengdu’s megacity dominance.

**Table 1:** Classification of city size levels in various economic regions of Sichuan Province.

Economic zones	Hierarchical scale	City names	Current situation
Chengdu Plain	Megacity	Chengdu	The Chengdu Plain Economic Zone concentrates over 50% of the province’s permanent population, representing the most developed, densely populated, and industrially concentrated region in Sichuan. It ranks among the most urbanized and economically agglomerated areas in Western China.
	Type II large city	Mianyang	
	Medium-sized city	Deyang, Suining, Leshan, and Meishan	
	Type I small city	Ziyang and Ya’an	
Northeastern Sichuan	Type II large city	Nanchong	The Northeastern Sichuan Economic Zone exhibits relatively underdeveloped economic conditions. Urban settlements across all hierarchical scales remain undersized, with regional centers Nanchong and Dazhou demonstrating limited radiating capacity. Certain towns and county seats face population outflow risks. The urbanization process lags behind provincial averages, constrained by infrastructure deficits and public service inadequacies.
	Medium-sized city	Dazhou	
	Type I small city	Guangyuan, Guang’an, and Bazhong	
Southern Sichuan	Type II large city	Yibin	The Southern Sichuan Economic Zone ranks second in provincial economic output. However, its core cities suffer from insufficient scale and weak agglomeration capacity, coupled with notable population outflow. The region also faces overlapping redundancies in core industries and public service provision.
	Medium-sized city	Zigong, Luzhou, and Neijiang	
Panxi	Medium-sized city	Panzhihua	The Panxi Economic Zone is currently the only state-approved experimental zone with the theme of comprehensive resource development and utilization. In terms of both resident population and regional GDP, it ranks fourth among the five major economic zones in Sichuan Province, indicating a relatively lagging overall development level.

#### 2.1.2 Data sources

This study uses both statistical data and web-based data. The statistical data used in this study were primarily sourced from the *Sichuan Statistical Yearbook 2023* (SSYB), *Sichuan Transportation Yearbook 2023* (STYB), municipal statistical yearbooks of individual cities (MSYB), and human resources and social security bulletins published by prefecture-level cities (MHRB). Data on licensed (assistant) physicians and hospital beds were mainly obtained from the *Sichuan Health Statistical Yearbook 2023* (SHSYB), while demographic indicators such as the proportion of population aged 65 and above (2020 data) were collected from the *Sichuan Population Census Yearbook 2020* (SPCY).

The infectious disease data pertains to the COVID-19 epidemic and was sourced from the official website of the Sichuan Provincial Health Commission.^1^ The dataset includes confirmed cases reported at the prefecture-level city scale, covering the period from January 1, 2020, to December 10, 2022.

The network data consists of Point of Interest (POI) data for prefecture-level cities in Sichuan Province in 2022, obtained from Amap (Amap POI)^2^ (22). This dataset includes the quantities of daily service facilities such as convenience stores, supermarkets, shopping malls, and restaurants, which are used to measure the density of living service venues. Additionally, the administrative boundary vector maps for each city were acquired from the National Platform for Common Geospatial Information Services.^3^ Table 2 summarizes the relevant data information, including data types, temporal resolution, time range, and data sources.

**Table 2:** Data sources.

Data type	Resolution	Time range	Data sources
Statistical yearbook data	Year	2022	https://tjj.sc.gov.cn/scstjj/c112132/pic_list.shtml
Point of Interest (POI) data	Year	2022	https://lbs.amap.com
Epidemic statistics data	Daily	January 1, 2020 – December 10, 2022	https://wsjkw.sc.gov.cn/
Administrative division boundary data			https://www.tianditu.gov.cn/

### 2.2 Infectious disease disasters comprehensive risk assessment index system

To comprehensively understand integrated disaster risk, international organizations such as the United Nations Office for Disaster Risk Reduction (UNDRR) and the United Nations Development Programme (UNDP) have incorporated disaster risk reduction measures into national planning and decision-making processes based on metric frameworks (23). The discourse on Disaster Risk Reduction (DRR) is undergoing a paradigm shift toward vulnerability-oriented approaches, with vulnerability emerging as a common evaluative characteristic in numerous risk assessments, providing practical information for accurate disaster prevention and mitigation (24).

Scholars have conducted in-depth research on integrated risks of infectious disease disasters. For instance: Mete et al. employed three risk factors from the INFORM COVID-19 Risk Index—hazard and exposure, lack of coping capacity, and vulnerability—to reassess national disaster risks in two phases (25); Pang et al. developed a disaster loss index model based on vector vulnerability, disaster-prone environmental instability, hazard intensity, disaster prevention capacity, and emergency response capability to study pandemic transmission’s environmental risks and socioeconomic impacts (26); Pluchino et al. established a risk index framework incorporating disease hazard (H), regional exposure (E), and population vulnerability (V) to assess epidemiological risks across geographical areas and identify high-risk zones (27); Kanga et al. created an integrated risk assessment framework combining hazard and vulnerability, defining infectious disease risk as C=H × V, followed by risk assessment and mapping (28). In summary, risk index evaluations primarily focus on disease risk, hazards, and vulnerability. By comprehensively considering multiple risk factors and their impacts, more effective risk assessment and management can be achieved. When constructing the comprehensive risk assessment index system for infectious disease disasters in this study, it becomes necessary to redefine these two subsystems—hazard and vulnerability.

The transmission intensity of infectious diseases determines both the likelihood of disease occurrence and the extent of its spread, necessitating the selection of indicators that can characterize disease transmission patterns as hazard factors. Analysis of viral epidemiological characteristics (29, 30) reveals that transmission routes primarily include aerosol transmission, airborne transmission, and direct contact transmission, with influencing factors being highly complex (31). The emergence and spread of infectious diseases are associated with several determinants, encompassing both anthropogenic factors (e.g., population density, travel and trade patterns, susceptibility across different demographic groups) and ecological factors (32). Therefore, this study extracts hazard-related influencing factors from the following dimensions: population characteristics (33), population aggregation (34), demographic dynamics (35), and environmental factors (36). These elements collectively form the framework for constructing the indicator system.

Epidemic disasters differ from natural disasters in that they primarily affect human health through interpersonal transmission and lead to lasting socioeconomic consequences. As a result, pandemic risk assessment studies tend to focus more on the vulnerability of populations and socioeconomic systems while often neglecting spatial considerations. However, many drivers of pandemic vulnerability are inherently linked to global connectivity and urbanization levels, arising from the complex interplay of spatial structural imbalances, uneven economic development, and insufficient governance capacity. Any deterioration in these factors may increase a city’s vulnerability and risk (37). In this study, we define vulnerability as the sensitivity of urban systems to external disturbances and their lack of coping capacity, which makes their structure and function prone to change.

To construct an urban vulnerability indicator system for public health emergencies, we conducted a comprehensive review of relevant literature, including the Population Vulnerability Index widely used in public health and medical fields (38), the Social Vulnerability Index (SVI) (39), urban vulnerability assessments (UVA) that incorporate both social and physical factors in local planning (40), and the Pandemic Vulnerability Index (PVI) (41). We extracted key influencing factors on urban vulnerability from socioeconomic (42), spatial-environmental (43), and infrastructural dimensions (44) to build our indicator system.

The establishment of a risk coupling assessment model for infectious disease hazards and urban vulnerability can effectively measure the threat level of infectious diseases and the degree of urban vulnerability, identify risk-influencing factors, and subsequently formulate targeted epidemic prevention and urban planning strategies. This provides crucial scientific support for disaster prevention and mitigation as well as resilient city development. To explore potential variables influencing infectious disease hazards and urban vulnerability, this study referenced variables included in previous research. Based on principles of data relevance, availability, and reliability, we screened and categorized key indicators to construct a comprehensive integrated risk assessment framework (Table 3).

**Table 3:** Comprehensive risk assessment indicator system for urban infectious disease disasters.

Target	Sub system	Dimension	Indicator	Definition	Source	Direction	Weight
Comprehensive risk	Infectious disease hazard	Population characteristics	Proportion of population aged 65 and over (X1)	Percentage of permanent residents aged ≥65 years in the region (%)	SPCY 2020	+	0.046
			Population living in poverty (X2)	Percentage of population receiving minimum living allowance (%)	MSYB2023	+	0.093
		Population agglomeration	Population Density (X3)	Permanent residents per unit land area (persons/km^2^)	SSYB 2023	+	0.135
			Employment Density (X4)	Total employed persons per built-up area (10,000 persons/km^2^)	MSYB2023	+	0.062
		Demographic dynamics	Domestic tourist arrivals (X5)	Annual domestic tourist arrivals (10,000 persons)	SSYB 2023	+	0.251
			Public transport vehicles per 10,000 population (X6)	Number of operational public buses/trolleys (urban districts) per 10,000 permanent residents (units/10,000 persons)	SSYB 2023	+	0.144
			Highway passenger traffic volume (X7)	Annual highway passenger transport volume (10,000 persons)	STYB 2023	+	0.120
		Environmental factors	PM₂.₅ concentration (X8)	Annual mean Air Quality Index (μg/m^3^)	SEEB2023	+	0.075
			Relative humidity (X9)	Annual mean relative humidity (%)	SSYB 2023	+	0.029
			Mean air temperature (X10)	Annual mean air temperature (°C)	SSYB 2023	−	0.044
	Urban vulnerability	Spatial vulnerability	Residential density (X11)	Per capita housing floor area (m^2^/person)	SSYB 2023	−	0.123
			Amenity density (X12)	Number of convenience stores/supermarkets/shopping malls per km^2^ (based on POI data)	Amap POI	+	0.072
			Transport facility density (X13)	Number of bus stops per km^2^ (based on POI data)	Amap POI	+	0.169
			Green coverage rate (X14)	Percentage of green space in built-up area (%)	SSYB 2023	−	0.054
			Open space density (X15)	Number of parks/public squares per km^2^	SSYB 2023	−	0.093
		Economic vulnerability	Unemployment rate (X16)	Registered urban unemployment rate (%)	MSYB2023	+	0.052
			Income per capita (X17)	Annual per capita disposable income of residents (10,000 CNY)	SSYB 2023	−	0.052
			Health expenditure as percentage of GDP (X18)	Government health expenditure as percentage of GDP (%)	SHSYB 2023	−	0.046
			Annual per capita household savings deposit balance (X19)	Per capita savings deposits of urban/rural residents (10,000 CNY)	SSYB 2023	−	0.049
			Emergency supplies reserve expenditure as a percentage of GDP (X20)	Government emergency reserves expenditure as percentage of GDP (%)	SSYB 2023	−	0.047
		Social vulnerability	Physicians per 10,000 people (X21)	Licensed (assistant) physicians per 10,000 permanent residents	SHSYB 2023	−	0.090
			Hospital beds per 10,000 inhabitants (X22)	Number of hospital beds per 10,000 permanent residents	SHSYB 2023	−	0.086
			Coverage rate of basic social security schemes (X23)	Coverage rate of basic pension insurance (%)	MHRB2023	−	0.068

### 2.3 Data standardization

In a multi-indicator evaluation system, different indicators may have varying units of measurement. Therefore, data standardization is required during the evaluation process. There are two types of evaluation indicators: positive and negative. For positive indicators, higher values indicate greater risk and vulnerability; for negative indicators, higher values indicate lower risk and vulnerability. Consequently, this study employs the extremum method to conduct positive transformation of all original indicators (Equations 1–2).

Positive indicators:

(1) $$X_{\mathrm{ij}}=\frac{x_{\mathrm{ij}}-x_{\min}}{x_{\max}-x_{\min}}(i=1,2\ldots ,m;j=1,2\ldots ,n)$$

Negative indicators:

(2) $$X_{\mathrm{ij}}=\frac{x_{\max}-x_{\mathrm{ij}}}{x_{\max}-x_{\min}}(i=1,2\ldots ,m;j=1,2\ldots ,n)$$

Where x_ij_ is the original data of the evaluation index; x_max_ and x_min_ are the maximum and minimum values of the evaluation index; X_ij_ is the indicator value after standardised processing. Here, i refers to the prefecture-level and above cities in the study, totaling m = 18. j represents the various indicators.

### 2.4 Entropy method

The entropy method objectively determines indicator weights by measuring information entropy to quantify data variability, effectively eliminating biases inherent in subjective weighting approaches (45). In information theory, entropy serves as a metric for system disorder and the amount of useful information contained within datasets. When evaluation objects demonstrate significant disparities in specific indicators, lower entropy values indicate greater informational utility, warranting higher weight assignments (46). The methodological procedure involves: standardizing raw data, calculating information entropy for each indicator, and deriving weight coefficients based on entropy values (47). This process rigorously accounts for relative importance among indicators, ensuring scientifically robust weight allocation. For public health risk assessment, the entropy method proves particularly effective in handling multi-source heterogeneous data, precisely capturing each risk factor’s actual contribution to support comprehensive evaluations (Equations 3–7).

The feature proportion of the i-th city under the j-th indicator can be defined as follows:

(3) $$P_{\mathrm{ij}}=\frac{X_{\mathrm{ij}}}{\sum_{i=1}^{m}X_{\mathrm{ij}}}$$

Where m represents the total number of prefecture-level and above cities (here, m = 18), and the calculation constant k is given by:

(4) $$k=\frac{1}{\ln(m)}$$

The Information entropy of the j-th indicator can be defined as follows:

(5) $$e_{j}=-k\sum_{i=1}^{m}P_{\mathrm{ij}}\ln(P_{\mathrm{ij}})$$

Calculate the divergence coefficient gj for the j-th indicator:

(6) $$g_{j}=1-e_{j}$$

Calculate the weight of the j-th indicator:

(7) $$w_{j}=\frac{g_{j}}{\sum_{j=1}^{n}g_{j}}$$

### 2.5 Measure the disaster hazard index and urban vulnerability index

This study calculates the disaster hazard index by combining standardized indicator values with their respective weights, reflecting both the hazard intensity levels and spatial distribution patterns across the study areas. The computational formula is expressed as follows (Equation 8):

(8) $$H_{i}=\sum_{j=1}^{n}W_{j}\times X_{\mathrm{ij}}$$

Where Hᵢ denotes the disaster hazard index for the i-th city, while higher values indicate greater hazard intensity; Wⱼ represents the weight of the j-th indicator derived from the entropy method, Xᵢⱼ corresponds to the standardized value of the indicator.

To quantify regional vulnerability, the same methodology was employed to calculate the urban vulnerability index, thereby enabling quantitative analysis of both the magnitude and spatial distribution of vulnerability across the study areas (Equation 9).

(9) $$V_{i}=\sum_{j=1}^{n}W_{j}\times X_{\mathrm{ij}}$$

Where Vᵢ denotes the urban vulnerability index for the i-th city, while higher values indicate greater vulnerability degree; Wⱼ represents the weight of the j-th indicator derived from the entropy method, Xᵢⱼ corresponds to the standardized value of the indicator.

### 2.6 Calculation of composite risk index

Risk analysis should concurrently consider both infectious disease hazard and urban vulnerability, as risk is a function of hazard and vulnerability. The computational formula can be expressed as (48, 49) (Equation 10).

(10) $$R_{i}=H_{i}\times V_{i}$$

The above calculation demonstrates that regional disaster risk escalates with increasing hazard intensity and vulnerability levels.

### 2.7 Coupling coordination degree model (CCDM)

The Coupling Coordination Degree Model (CCDM), based on coupling theory, effectively evaluates interaction effects and coordinated development levels between different systems. It has been widely applied to examine relationships among social, economic, and ecological systems (50, 51), including: production-living-ecological spaces (52, 53), economy-ecology interplay (54, 55), Urbanization-ecological environment dynamics (56), Cultural landscape conservation vs. socioeconomic development (57). Recently, CCDM has transitioned from social-economic-ecological studies to disaster risk research, enabling in-depth analyses of: spatial coupling relationship between multidimensional poverty and the risk of geological disaster (58), the coupling relationship between flood risk and population vulnerability (59), integrated effects and multidimensional impacts of “Hazard-Exposure-Vulnerability” on urban flood risks (60). These studies demonstrate applicability of CCDM in disaster risk assessment frameworks. However, existing research lacks spatial coupling perspectives to unravel interaction mechanisms between acute public health hazards and urban vulnerability.

“Coupling” refers to the process of interaction and mutual influence between two or more elements (61). This study employs CCDM to analysis the interdependent or mutually constraining relationships between disaster hazards and urban vulnerability. Within CCDM research, most scholars adopt the conventional model structure, calculated as follows (Equations 11–13):

(11) $$C=2\times \sqrt{\frac{H_{i}\times V_{i}}{{\left(H_{i}+V_{i}\right)}^{2}}}$$

(12) $$T=\alpha H_{i}+\beta V_{i}$$

(13) $$D=\sqrt{C\times T}$$

Given the dimensional differences between disaster hazards and urban vulnerability, normalized ordinal values were employed to calculate their synchronization and overall coordination degree (Equations 14–16). Based on the final coupling coordination degree (D) values, and referencing the classification framework from Xiang et al.’s study (58), the coordinated development status between disaster hazards and urban vulnerability was categorized into 4 major classes. These were further subdivided into 6 subtypes according to the proportional relationship between the two systems (Table 4).

(14) $$C=\frac{f{\left(x\right)}^{k}\times g{\left(x\right)}^{k}}{{\left(\mathrm{\alpha f}(x)+\mathrm{\beta g}(x)\right)}^{2k}}$$

(15) $$T=\sqrt{\mathrm{\alpha f}(x)\times \mathrm{\beta g}(x)}$$

(16) $$D=\sqrt{C\times T}$$

**Table 4:** Classification of coupling coordination types between disaster hazard and urban vulnerability.

Coupling coordination type	Coupling coordination degree	Classification rule	Relation discrimination feature	Coupling coordination subtype
Coordinated development	0.6 ≤ D ≤ 1	0 ≤ |H—V| ≤ 0.1	Synchronized coordinated development	Synchronization development
		H—V > 0.1	Coordinated development with urban vulnerability lag	Coordinated-urban vulnerability lagging
		V—H > 0.1	Coordinated development with disaster hazard lag	Coordinated-disaster hazards lagging
Barely coordinated development	0.5 ≤ D<0.6	0 ≤ |H—V| ≤ 0.1	Synchronised barely coordinative development	Synchronization development
		H—V > 0.1	Barely coordinated development with urban vulnerability lag	Coordinated-urban vulnerability lagging
		V—H > 0.1	Barely coordinated development with disaster hazards lag	Coordinated-disaster hazards lagging
On the verge of disorder	0.4 ≤ D<0.5	0 ≤ |H—V| ≤ 0.1	Synchronised on the verge of disorder	Disorder of both hazards and vulnerability
		H—V > 0.1	On the verge of disorder development with urban vulnerability lag	Disorder-urban vulnerability lag
		V—H > 0.1	On the verge of disorder development with disaster hazards lag	Disorder-disaster hazards lag
Disorder and recession	0 ≤ D<0.4	0 ≤ |H—V| ≤ 0.1	Synchronised disorder and recession development	Disorder of both hazards and vulnerability
		H—V > 0.1	Disorder and recession Development with urban vulnerability lag	Disorder-urban vulnerability lag
		V—H > 0.1	Disorder and recession Development with disaster hazards lag	Disorder-disaster hazards lag

Where C is the coupling degree, T is the coordination index between disaster hazards and urban vulnerability, D is the coupling coordination degree. f(x) is the normalised value of disaster hazards ranking; g(x) is the normalised value of urban vulnerability ranking. k is an adjustment coefficient (typically 2 ≤ k ≤ 5). To enhance discriminative capacity, this study sets k = 3 following Su et al. (62). Considering that f(x) is as important as g(x) (i.e., α + β = 1, with α = β = 0.5). The higher the value of D is, the better the coordination degree between disaster hazards and urban vulnerability is.

## 3 Results

### 3.1 Integrated measurement and spatial distribution of infectious disease hazard and urban vulnerability

**Figure 2:**
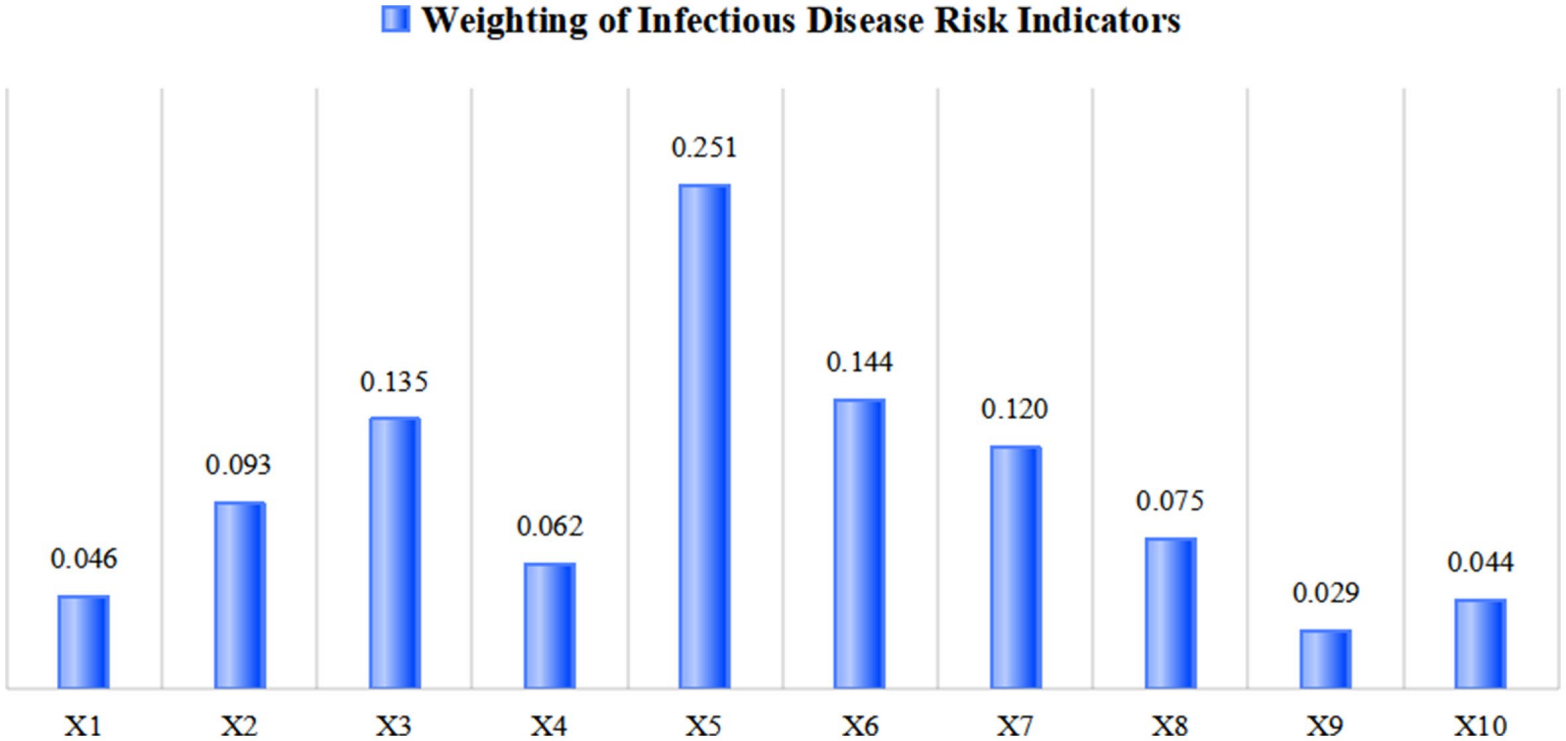
Weight of infectious disease hazard indicators.

**Figure 3:**
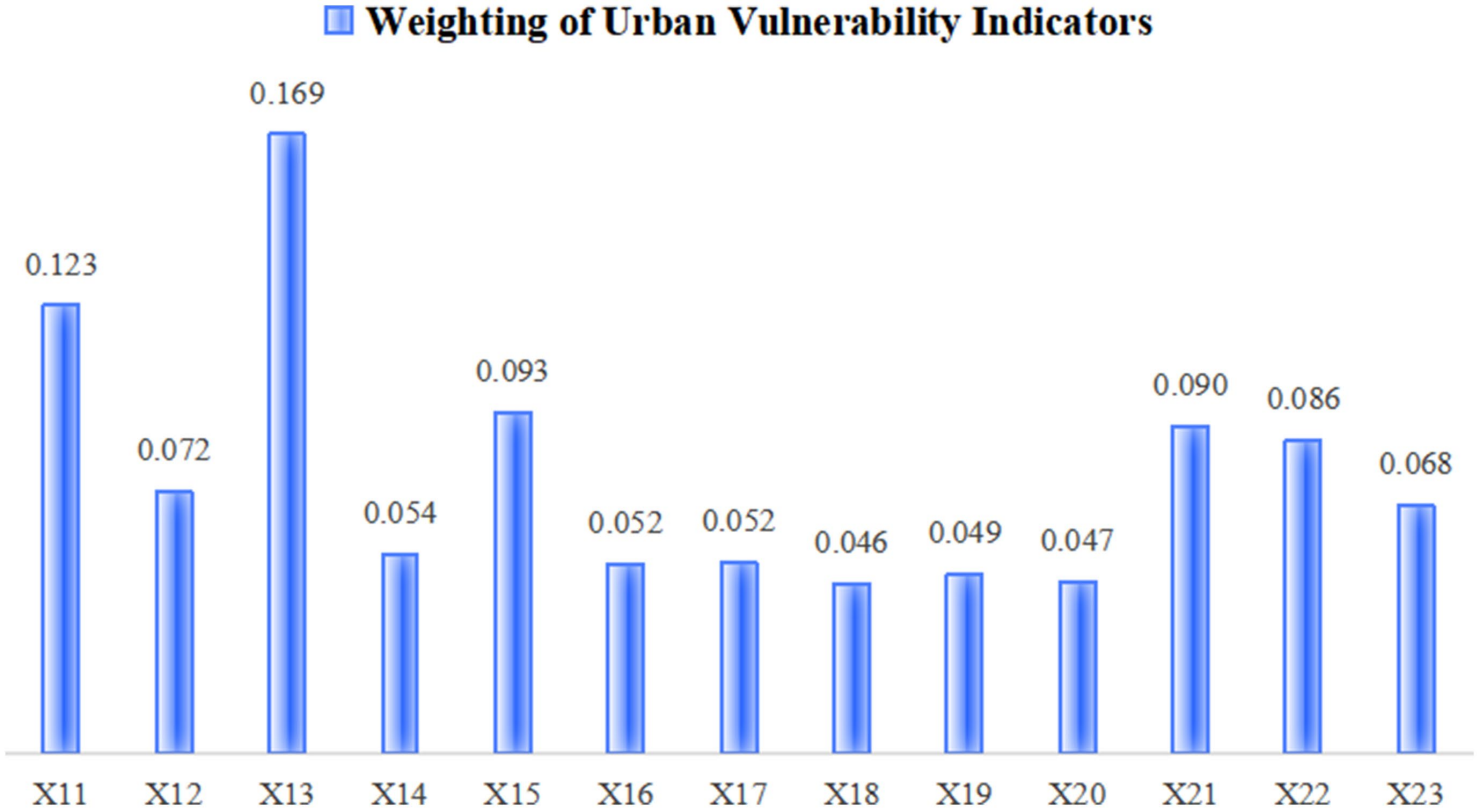
Weight of urban vulnerability indicators.

The standardized indicators were objectively weighted using the entropy method, yielding the respective indicator weights for infectious disease hazards and urban vulnerability (Figures 2, 3) as well as dimensional indices (Table 3). This enabled quantitative measurement of infectious disease hazards, urban vulnerability, comprehensive risk, and coupling coordination degree, with regional distribution patterns visualized through spatial mapping techniques. Furthermore, the study conducted qualitative analysis by incorporating regional development characteristics specific to Sichuan Province.

#### 3.1.1 Comprehensive measurements and spatial distribution of the infectious disease hazard

Through a comprehensive evaluation of population characteristics, population aggregation, population mobility, and environmental exposure, this study reveals the infectious disease risk levels and spatial distribution patterns across cities in Sichuan Province. The quantitative risk scores ranged from 0.149 to 0.761. Chengdu exhibited the highest risk index at 0.761, while all other cities scored below 0.5, indicating generally low-to-moderate risk levels. These findings demonstrate the significant effectiveness of Sichuan’s regional epidemic prevention policies in risk management.

**Figure 4:**
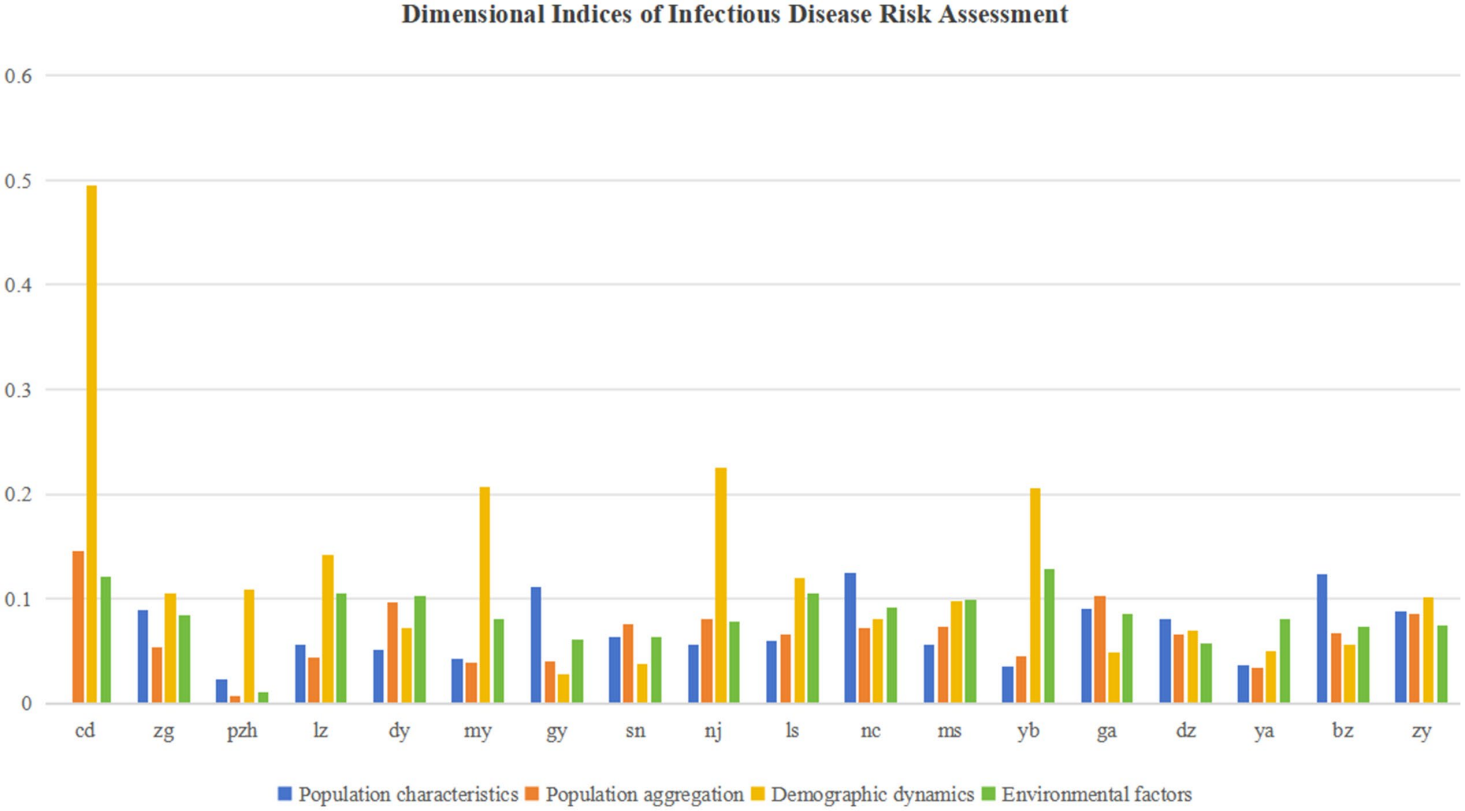
Dimensional indices of infectious disease hazard.

Furthermore, Table 5 and Figure 4 show that population mobility constitutes the most influential factor for infectious disease risk. The key contributing elements include domestic tourist numbers, public transportation vehicles per 10,000 people, population density, and highway passenger volume.

**Table 5:** Integrated risk assessment: subsystem components and dimensional indexation.

Cities	Population characteristics	Population aggregation	Demographic dynamics	Environmental factors	Infectious disease hazard	Spatial Vulnerability	Economic Vulnerability	Social Vulnerability	Urban Vulnerability	Comprehensive risk
cd	0.001	0.145	0.495	0.121	0.761	0.233	0.091	0.059	0.383	0.291
zg	0.089	0.054	0.105	0.084	0.331	0.161	0.193	0.061	0.415	0.138
pzh	0.023	0.007	0.109	0.010	0.149	0.204	0.180	0.079	0.464	0.069
lz	0.056	0.044	0.142	0.105	0.348	0.187	0.163	0.119	0.469	0.163
dy	0.051	0.097	0.072	0.103	0.322	0.314	0.146	0.120	0.580	0.187
my	0.043	0.040	0.207	0.080	0.369	0.253	0.160	0.120	0.533	0.197
gy	0.111	0.040	0.028	0.061	0.240	0.185	0.160	0.073	0.419	0.101
sn	0.064	0.076	0.038	0.063	0.240	0.177	0.199	0.145	0.521	0.125
nj	0.057	0.081	0.225	0.078	0.441	0.288	0.195	0.102	0.585	0.258
ls	0.059	0.066	0.119	0.105	0.351	0.292	0.183	0.114	0.590	0.207
nc	0.125	0.072	0.080	0.091	0.369	0.076	0.188	0.138	0.402	0.148
ms	0.056	0.073	0.098	0.100	0.327	0.401	0.163	0.156	0.720	0.235
yb	0.035	0.045	0.205	0.129	0.414	0.219	0.191	0.156	0.566	0.234
ga	0.091	0.103	0.049	0.085	0.327	0.286	0.172	0.200	0.659	0.216
dz	0.081	0.065	0.069	0.057	0.273	0.148	0.181	0.180	0.508	0.139
ya	0.037	0.034	0.049	0.080	0.201	0.285	0.187	0.041	0.513	0.103
bz	0.124	0.067	0.056	0.073	0.320	0.247	0.136	0.135	0.518	0.166
zy	0.088	0.086	0.102	0.074	0.350	0.336	0.185	0.087	0.608	0.213

**Figure 5:**
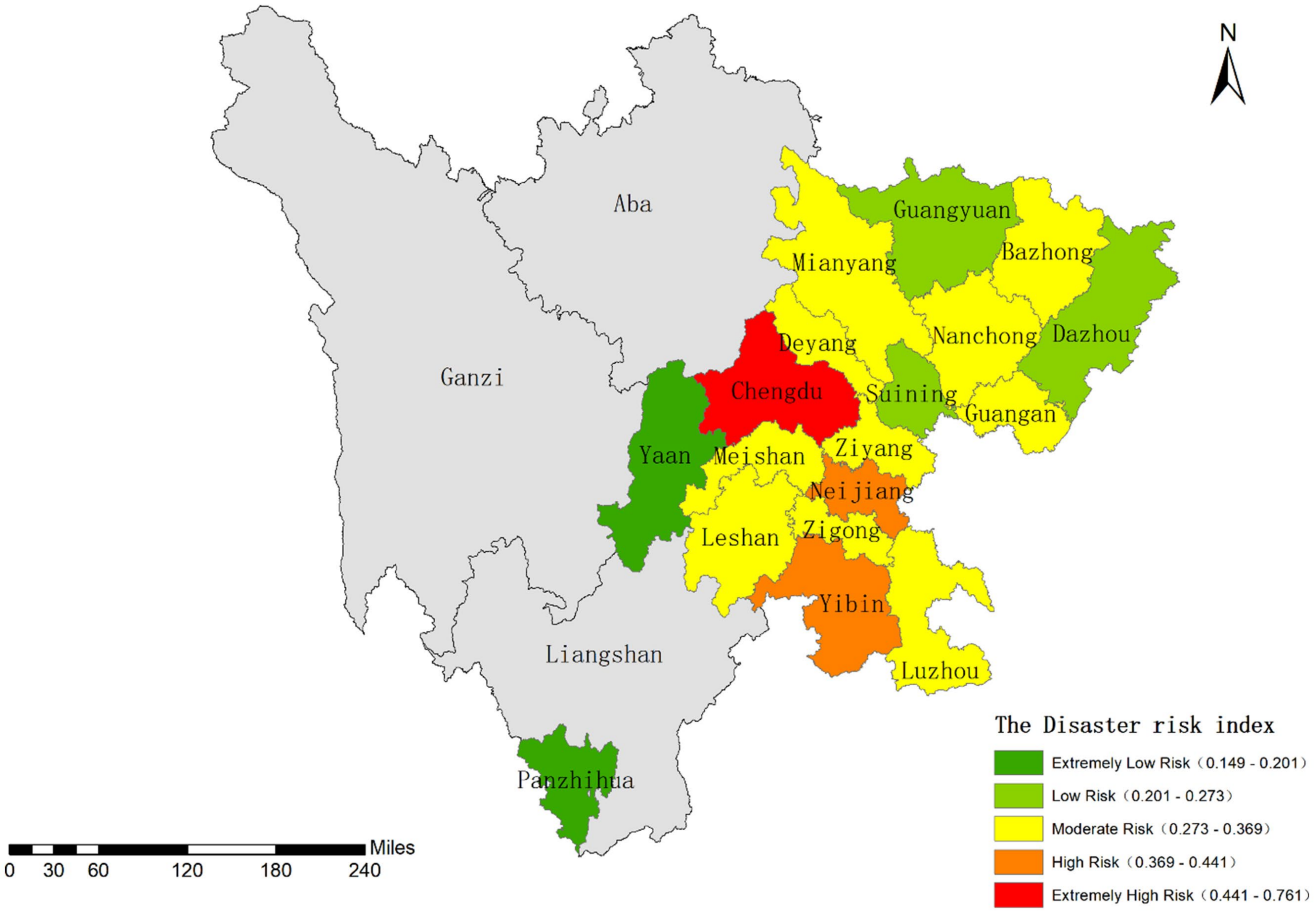
Classification results of the disaster hazard index.

Using the Natural Breaks method in ArcGIS (63, 64), the hazard index was classified into five risk levels: extremely high, high, moderate, low, and very low (Figure 5). Spatially, the disaster risk across Sichuan Province exhibits distinct regional differentiation, closely correlated with urban scale, geo-economic factors, and natural environment.

The extremely high-risk zone is represented by the megacity Chengdu, where elevated risk likely stems from dense population, economic activities, and urban expansion-induced environmental disturbances. Medium-to-high risk zones include large and medium-sized cities such as Yibin, Luzhou, Leshan, Meishan, and Zigong. In contrast, low and very low-risk areas are primarily distributed across smaller peripheral cities like Panzhihua, Guangyuan, and Dazhou, where abundant environmental resources and lower development intensity may contribute to risk mitigation.

Overall, this spatial risk pattern reflects both the constraints of natural geographical conditions and the impacts of regional development disparities.

#### 3.1.2 Comprehensive measurements and spatial distribution of the urban vulnerability

The urban vulnerability index reflects a city system’s sensitivity to internal and external disturbances and its lack of coping capacity—attributes that make its structure and function prone to change. Through a comprehensive evaluation of spatial layout, economic development, and social systems, this study reveals the vulnerability levels and spatial distribution characteristics across cities in Sichuan Province.

Quantitative vulnerability scores ranged from 0.383 to 0.720. Chengdu showed the lowest vulnerability index (0.383), followed by Nanchong (0.402), while most other cities scored above 0.5, indicating medium-to-high vulnerability levels. Meishan exhibited the highest vulnerability index at 0.720. These results demonstrate the inherent vulnerability of urban systems in Sichuan when responding to public health emergencies.

**Figure 6:**
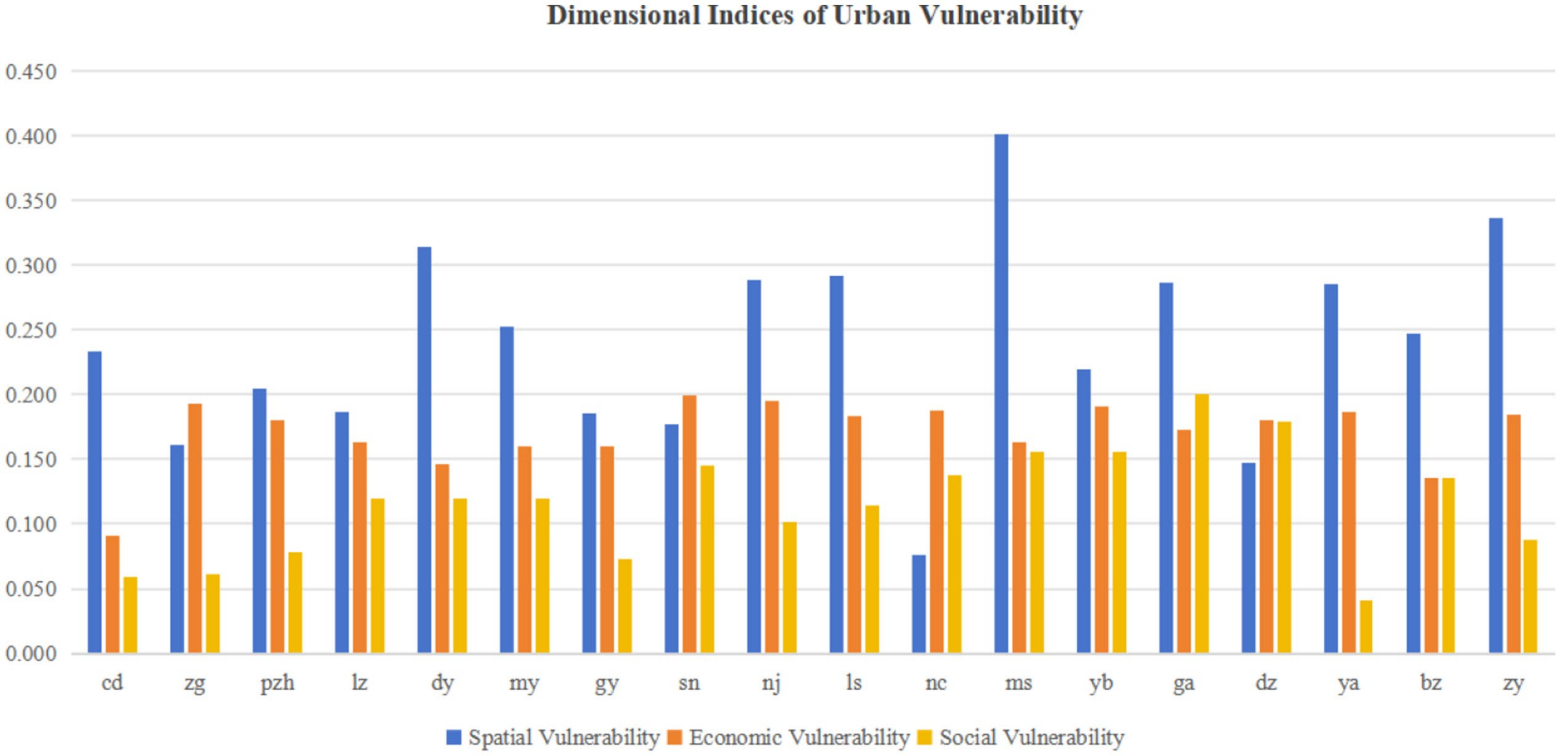
Dimensional indices of urban vulnerability.

As shown in Table 5 and Figure 6, spatial vulnerability demonstrated the most significant average influence. Key contributing factors included transportation facility density, residential density, road network density, open space density, number of doctors, and hospital bed capacity. This reflects the close relationship between regional development levels and the balance of spatial configurations.

**Figure 7:**
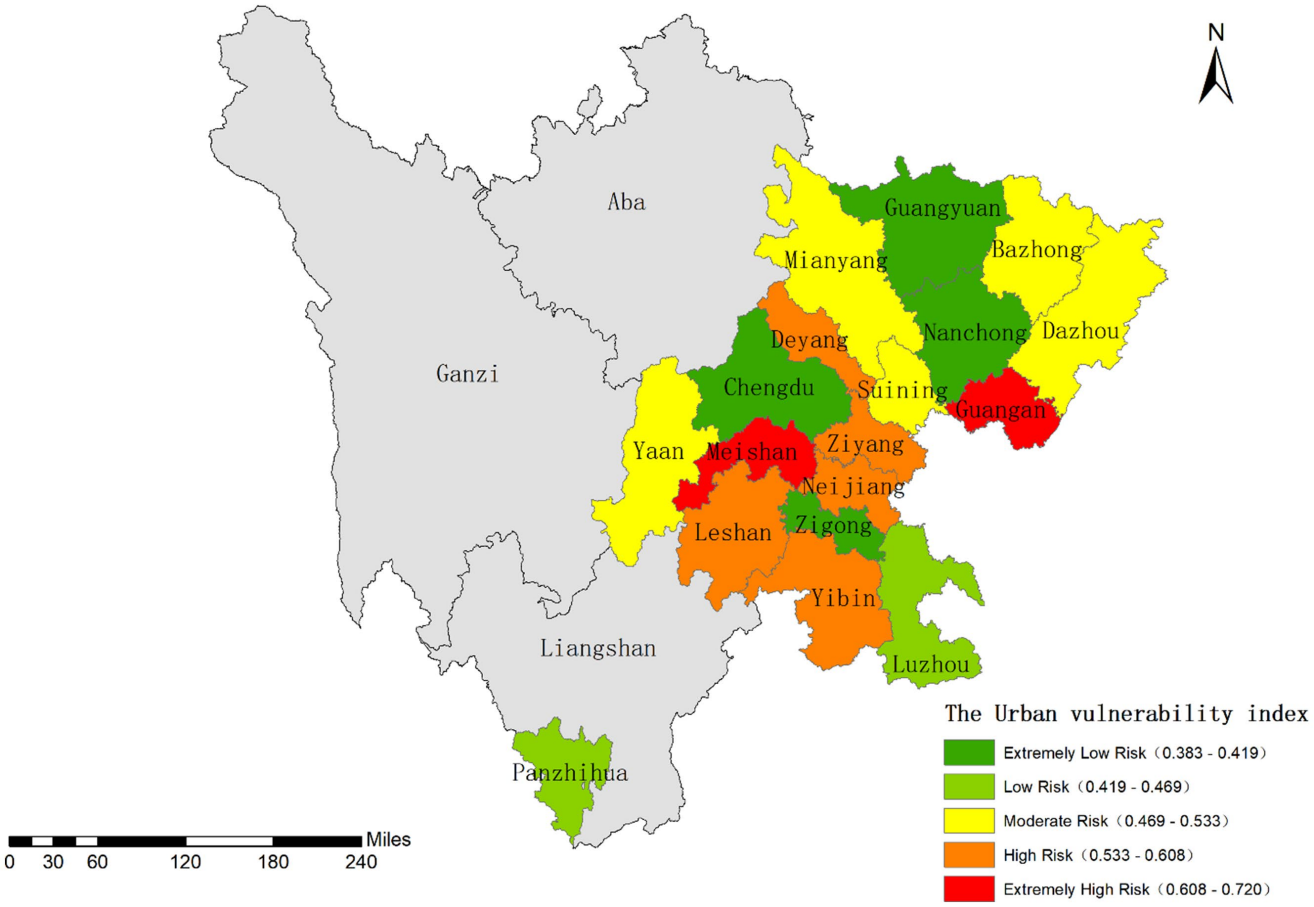
Classification results of the urban vulnerability index.

Using the Natural Breaks classification method in ArcGIS (63, 64), the vulnerability index was categorized into five levels: extremely high, high, moderate, low, and extremely low vulnerability zones (Figure 7). Spatially, urban vulnerability across Sichuan Province exhibits distinct regional differentiation.

The extremely low vulnerability zone is represented by the megacity Chengdu, whose resilience stems from advanced economic development and a comprehensive public service system. Cities like Nanchong and Zigong also demonstrate relatively low vulnerability due to their sound economic conditions, while Guangyuan benefits from stable geological environmental conditions.

Panzhihua and Luzhou fall into the low vulnerability category, with Mianyang and Suining classified as moderately vulnerable. High vulnerability areas such as Leshan, Yibin, and Guang’an may be influenced by multiple factors including geological disaster risks, industrial environmental pressures, and imbalanced public services.

Overall, this spatial pattern of urban vulnerability reflects both variations in natural geographical conditions and disparities in regional development levels.

### 3.2 Comparative analysis of composite risk mapping versus observed epidemic risk patterns

Based on the aforementioned assessment results of infectious disease hazards and urban vulnerability, we calculated the comprehensive risk index. Sichuan Province generally exhibits a low overall risk level. The provincial capital Chengdu showed the highest risk index (0.291), while Guangyuan and Panzhihua demonstrated the lowest values.

**Figure 8:**
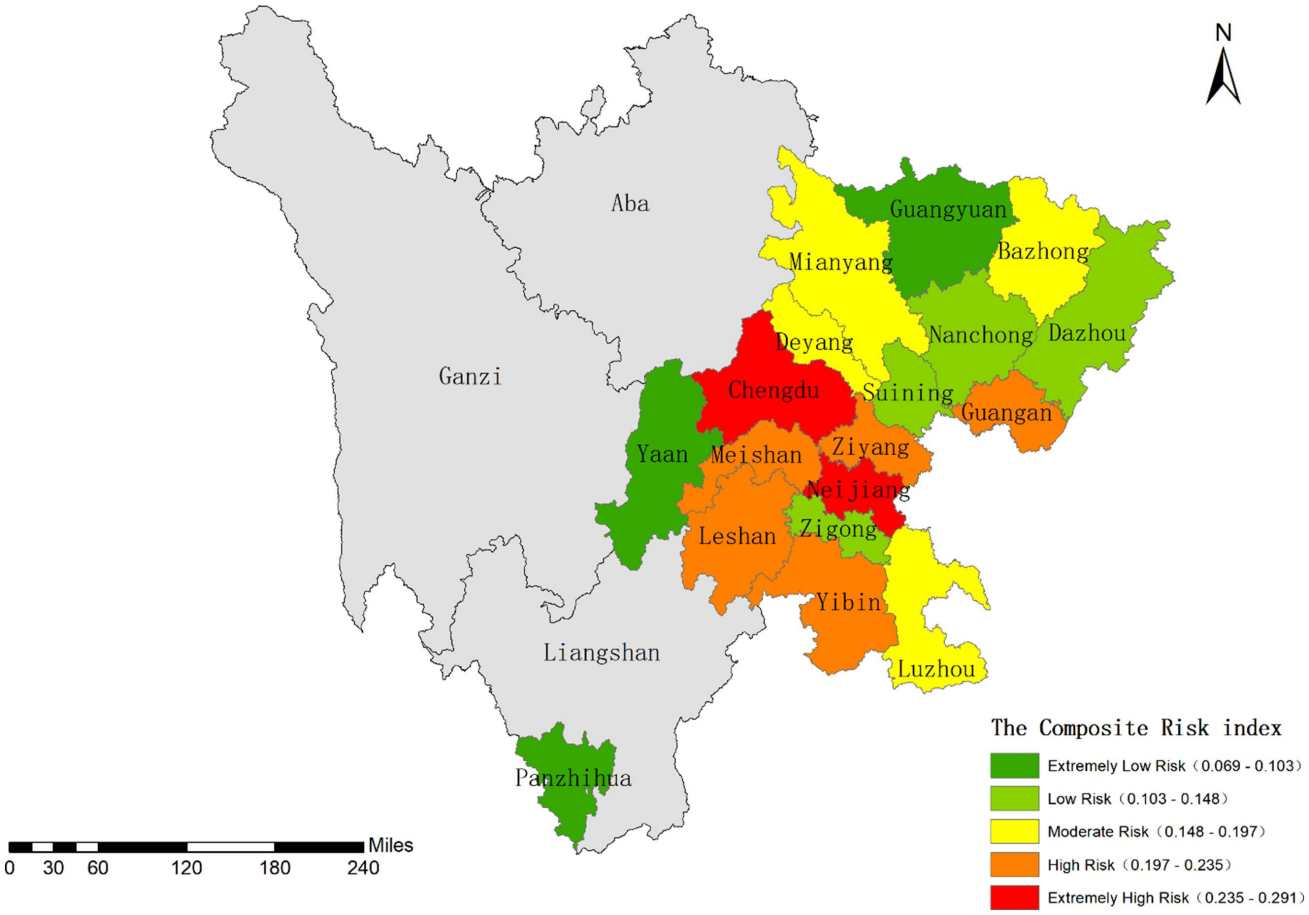
Classification results of the composite risk index.

Using the Natural Breaks classification method in ArcGIS (63, 64), the study area was divided into five risk levels: low, relatively low, moderate, relatively high, and high risk zones, as illustrated in Figure 8. Spatially, the risk distribution across Sichuan follows a “high in central regions, low in peripheral areas” pattern. The Chengdu Plain and southern Sichuan regions show significantly higher risk levels compared to Panxi and northeastern Sichuan areas.

The Chengdu Plain and southern Sichuan concentrate most of the province’s large and medium-sized cities. While these urban areas benefit from advanced economic development and well-established infrastructure, they simultaneously face challenges of high population density and strong mobility, leading to elevated infectious disease transmission risks. Notably, Chengdu presents a characteristic “low vulnerability-high risk” profile. In contrast, Panxi and northeastern Sichuan primarily consist of smaller cities or remote areas (e.g., Panzhihua, Guangyuan, and Ya’an), featuring lower population density, reduced human activity intensity, and higher natural environmental carrying capacity, resulting in comparatively lower comprehensive risk indices.

We conducted correlation analysis between the comprehensive risk index and actual cumulative infection cases to evaluate whether this risk indicator system could effectively reflect infection patterns (65). Spearman’s rank correlation analysis (Table 6) revealed a statistically significant positive correlation between the two variables (rs = 0.680, *p* = 0.002) at the 0.01 significance level. These results demonstrate that: increased comprehensive risk index significantly correlates with higher cumulative infection numbers; and the geographical distribution characteristics of the risk index effectively mirror the spatial distribution patterns of actual case numbers.

**Table 6:** Spearman’s rank correlation analysis.

Correlation				
			The composite risk index	Actual cumulative case counts
Spearman’s rho	The composite risk index	Correlation coefficient	1.000	0.680**
		Significance (two-tailed)		0.002
		*N*	18	18
	Actual cumulative case counts	Correlation coefficient	0.680**	1.000
		Significance (two-tailed)	0.002	
		*N*	18	18

** The correlation is statistically significant at the 0.01 level (two-tailed).

### 3.3 Measurement results and spatial characteristics of CCD

This study calculated the Coupling Coordination Degree (CCD) index to examine the interaction and coordination between infectious disease hazards and urban vulnerability. The overall CCD level across the study area was relatively low (mean = 0.384). Guangyuan and Panzhihua showed the highest CCD values (0.655 and 0.649, respectively), while Chengdu had the lowest CCD (0.031). As a megacity, Chengdu exhibited extremely low urban vulnerability but exceptionally high infectious disease risk, resulting in severe coupling coordination imbalance.

**Figure 9:**
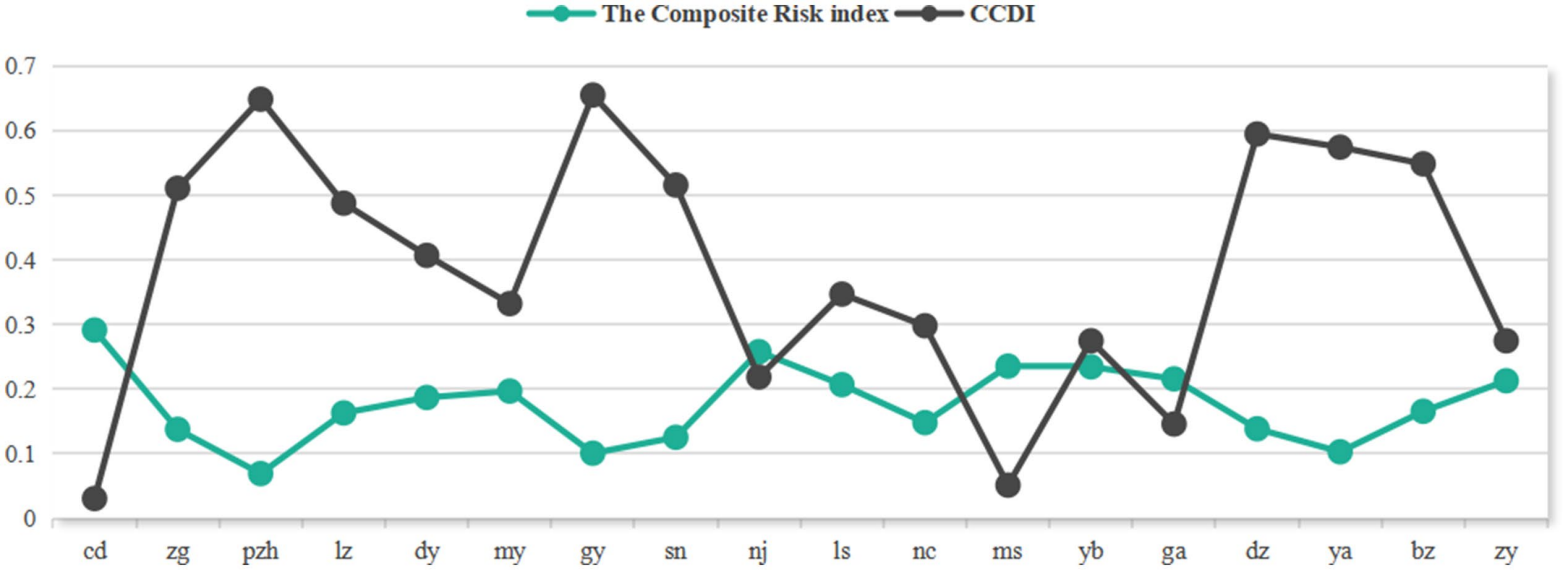
Contrast diagram between risk index and coupling coordination degree.

**Figure 10:**
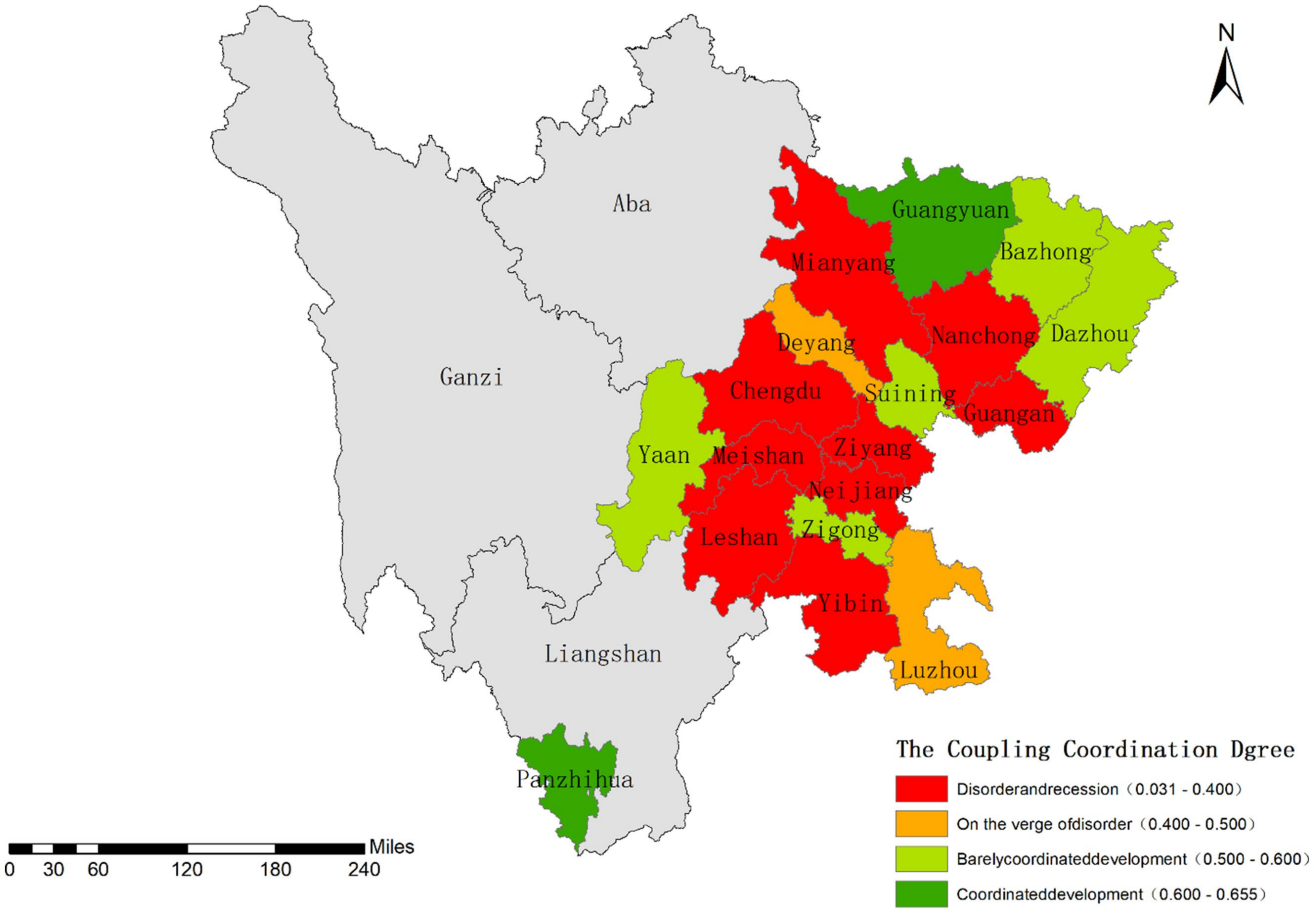
Distribution characteristics of the coupling coordination degree between disaster risk and urban vulnerability.

Based on the CCD model established in Section 2.7, we classified the study area into four coordination types using the following thresholds: coordinated development (0.6 ≤ D ≤ 1), marginal coordination (0.5 ≤ D < 0.6), near imbalance (0.4 ≤ D < 0.5), and declined imbalance (0 ≤ D < 0.4). Figure 9 reveals a spatial pattern where “lower CCD corresponds to higher risk.” Coordinated development cities were spatially dispersed, whereas declined imbalance cities were predominantly concentrated in the Chengdu Plain and southern Sichuan regions (Figure 10).

In-depth analysis reveals that the “hazard-dominant lagging” pattern predominates in the coupling coordination types across Sichuan Province. Cities achieving coordinated or marginally coordinated development, such as Panzhihua, Zigong, and Guangyuan, have made preliminary progress in aligning risk governance with spatial planning. Within the disordered and declined coordination categories, the regional risk patterns exhibit significant spatial heterogeneity: Chengdu demonstrates a characteristic “imbalanced-vulnerability lagging” type, while other cities predominantly follow an “imbalanced-hazard lagging” pattern. Empirical evidence confirms a notable positive feedback effect between urban vulnerability levels and infectious disease hazards, where heightened vulnerability not only exacerbates epidemic transmission risks by weakening systemic resilience but also perpetuates coupling system imbalances.

The aforementioned research reveals that the core mechanism influencing coupled coordinated development stems from multidimensional interactions among economic-spatial-social-environmental systems. This manifests in two distinct patterns: In rapidly developing regions (e.g., Chengdu, Mianyang), economic growth leads to excessive factor concentration, forming a “high investment-high density-high risk” transmission chain; whereas in underdeveloped areas (e.g., Guang’an), insufficient development momentum creates a vicious cycle of “low output-low protection-high vulnerability.” These findings align with the “economic foundation-spatial structure-governance capacity” synergistic mechanism proposed in Shekhar et al. (37), unveiling the coupled pathways of regional system complexity:

First, the economic-spatial coupling in the Chengdu Plain region demonstrates a significant positive feedback effect. Taking Chengdu and Mianyang as examples, the agglomeration of high-tech industries has driven population concentration and mobility (66), while simultaneously triggering land-use pattern restructuring. However, excessive intensive development has led to a decline in per capita public service resources (34). This “high-density, high-pressure” coupling model resulted in infection risks in core urban areas far exceeding surrounding regions during the pandemic, confirming the risk accumulation effects brought about by economically-driven spatial restructuring.

Second, the economic-social coupling in northeastern Sichuan manifests as a bidirectional inhibitory effect. A representative case was the “May 09” outbreak in Guang’an, where monthly infections surged to 1,299 cases. This episode revealed that the region’s per capita medical expenditure lagged behind the provincial average, with medical facility shortages directly accelerating epidemic spread. The event not only demonstrated how infrastructure vulnerability amplifies risk transmission, but more importantly exposed the underlying mechanism: sluggish economic development severely constrains public service investment (67), while inadequate social protection simultaneously restricts human capital development and suppresses economic growth by weakening consumption capacity.

Third, the economic-environmental coupling demonstrates distinct stress effects in industrial cities, particularly evident in Nanchong, Yibin, and Meishan. The expansion of traditional industries (e.g., liquor manufacturing in Yibin, textile and chemical production in Nanchong) has driven increased PM2.5 concentrations and water quality deterioration. Insufficient environmental protection investment further exacerbates health risks, empirically validating how the tension between development intensity and ecological carrying capacity translates into public health threats.

Fourth, the coupling coordination between spatial and economic systems is particularly prominent in Panzhihua. Through measures including intensive mining area redevelopment, functional diversification in central urban zones, and strict ecological conservation management, the city has achieved optimal alignment between spatial resource allocation and economic development needs. This spatial optimization strategy has established Panzhihua as a core city in China’s national comprehensive resource utilization pilot zone. Such a coordinated development pathway provides replicable practical solutions for peer cities to construct more resilient industry-space-ecological systems.

Fifth, the synergistic effects of space-society-environment systems are prominently demonstrated in Guangyuan City. As Sichuan Province’s highest CCD-scoring city (CCD = 0.655), Guangyuan has achieved virtuous interaction between social systems and environmental governance through its “low-density, high-investment” development model. Leveraging its mountainous topography to create a polycentric spatial configuration, the city maintains relatively low population density while compensating for economic development limitations through extraordinary healthcare resource allocation and robust social security systems. This governance approach—integrating decentralized spatial structures, premium ecological endowments, and targeted social investments—has not only significantly reduced epidemic transmission risks but also established a distinctive resilient development paradigm for mountainous cities. It provides valuable reference for coordinating socioeconomic development with ecological conservation in underdeveloped western regions.

These coupling mechanisms not only deconstruct the generative logic of risk heterogeneity, but also empirically validate through typical cases a transmission chain of: industrial agglomeration (economic) → land-use compactness (spatial) → service coverage (social) → ecological sensitivity (environmental). This provides a differentiated theoretical framework for regional resilience planning.

## 4 Discussion and research contributions

### 4.1 Discussion

The coordinated development between infectious disease hazards and urban vulnerability constitutes a critical component of urban public health risk governance (68). These two systems interact through factor flows and feedback mechanisms, collectively determining regional comprehensive risk levels. This study has measured infectious disease hazards, urban vulnerability, comprehensive risks, and coupling coordination, with regional distribution patterns visualized through spatial mapping techniques.

#### 4.1.1 Validation of the CCD model

**Figure 11:**
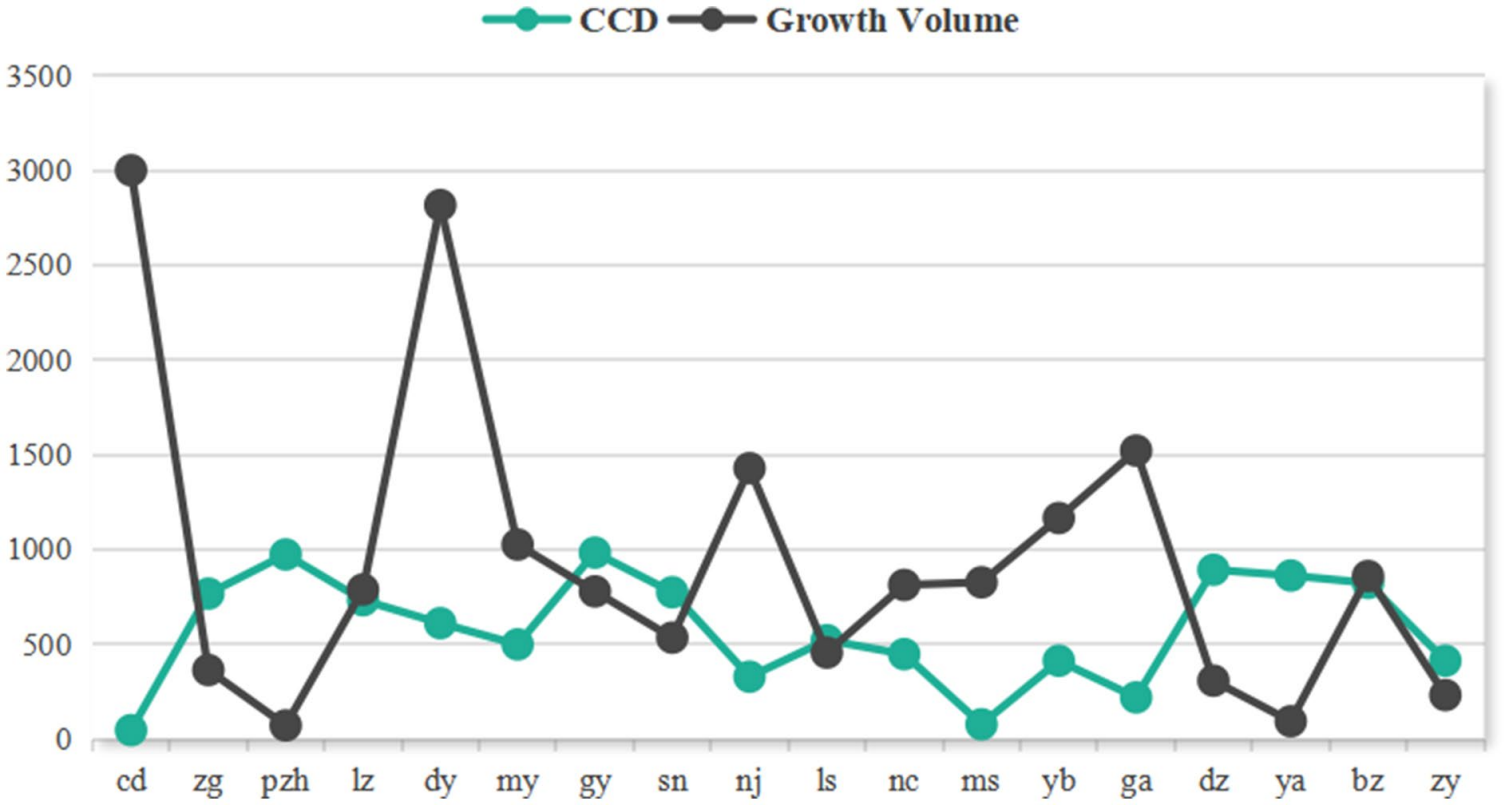
Comparison diagram of coupling coordination degree and case growth volume.

This study validated the coupling coordination degree (CCD) assessment results using cumulative infection case data from prefecture-level cities in Sichuan Province between January 1, 2020 and December 31, 2021. The case growth increment (ΔC = C_2022_ – C_2020-2021_) was employed for verification, generating ΔC and CCDI zonal statistics maps for each city (Figure 11—the Y-axis values of CCD were magnified for detail display, while case increment data were compressed to a 3,000-range display, though Chengdu’s values remained the provincial maximum).

The results show that Chengdu exhibited the highest case growth increment (16,341 cases) in the province, far exceeding other cities, which corresponds with its lowest CCDI (0.031) in Sichuan. In striking contrast, Panzhihua recorded the lowest case growth increment (71 cases), demonstrating an inverse relationship with its high CCD index (0.649).

The research findings demonstrate a significant negative correlation between epidemic development and coupling coordination degree (CCD) across Sichuan Province’s cities during the Omicron variant outbreak. Cities with higher CCD values (e.g., Panzhihua) exhibited stronger epidemic resistance capabilities, maintaining relatively low case growth increments. Conversely, cities with lower CCD values (e.g., Chengdu) faced substantially greater epidemic prevention and control pressures. These results indicate that the CCD model can effectively predict case number trends across cities, validating the model’s accuracy and applicability in assessing regional epidemic prevention efficacy (69). The findings provide scientific evidence for formulating differentiated prevention and control strategies.

#### 4.1.2 Comparative analysis with conventional risk models

The comparative analysis between the CCD model and traditional risk assessment models (Table 7) demonstrates that the CCD framework transcends the linear paradigms of conventional approaches (e.g., weighted summation or multiplicative models). Its fundamental innovation lies in capturing dynamic feedback mechanisms while quantitatively characterizing the synergistic interactions between hazards and vulnerability through coupling degree (C) and coordination degree (T), enabling the identification of vicious cycles such as accelerated disease transmission resulting from medical resource depletion (70). By leveraging the spatial heterogeneity characteristics of coupling coordination degree (D), the model overcomes the coarse-scale limitations inherent in regional averaging methods, thereby facilitating differentiated interventions across cities (71). Moreover, the analysis of coordination lag types (hazard-dominant versus vulnerability-dominant) facilitates the mapping of priority measures, effectively circumventing the resource misallocation associated with traditional homogeneous high-score interventions (72). Consequently, the coupling coordination degree model has emerged as a robust analytical tool for evaluating balanced regional development.

**Table 7:** Comparative analysis of CCD model vs. traditional models.

Dimension	Traditional models (INFORM, PVI)	CCD model	Comparative framework
Risk composition	Linear additivity:	Nonlinear coupling:	Captures synergistic interactions between hazard and vulnerability (Synergistic Interaction)
	$R_{i}=\sum_{j=1}^{n}W_{j}\times X_{\mathrm{ij}}$	$C=2\times \sqrt{\frac{H_{i}\times V_{i}}{{\left(H_{i}+V_{i}\right)}^{2}}}$ $T=\alpha H_{i}+\beta V_{i}$ $D=\sqrt{C\times T}$	
	$R_{i}=H_{i}\times V_{i}$	$C=\frac{f{\left(x\right)}^{k}\times g{\left(x\right)}^{k}}{{\left(\mathrm{\alpha f}(x)+\mathrm{\beta g}(x)\right)}^{2k}}$ $T=\sqrt{\mathrm{\alpha f}(x)\times \mathrm{\beta g}(x)}$ $D=\sqrt{C\times T}$	
Mechanism	Unidirectional Causality (e.g., Vulnerability → Risk)	Bidirectional Feedback Loops (e.g., Medical surge → Transmission acceleration → ↑Vulnerability)	Deciphers dynamic systemic risk circuits (Dynamic feedback mechanisms)
Output	Static risk scores	Coordination typology + Lag direction (e.g., Hazard-lagged/Vulnerability-lagged)	Identifies priority domains for risk governance (Spatial heterogeneity)
Policy recommendations	Homogeneous Intervention via Static Zoning: Administrative unit classification → Standardized policy deploymen	Heterogeneous Leverage Regulation via Dynamic Feedback: Feedback diagnosis → Targeted governance	Transforms passive response to proactive risk shaping, enabling context-sensitive strategies (Contextual factors)

### 4.2 The main contributions of this study

This study developed a “hazard-vulnerability” risk coupling model based on the entropy method and Coupling Coordination Degree (CCD) model, systematically analyzing coupling coordination mechanisms and key influencing factors. The results reveal that the regional risk coupling system exhibits a distinct “high in central areas, low in peripheral regions” spatial differentiation pattern, where economic vulnerability, spatial vulnerability, and social vulnerability demonstrate complex nonlinear interactions.

To verify the reliability of research conclusions, this study conducted systematic validation through a triple-verification approach: First, correlation analysis between cumulative confirmed cases and the comprehensive risk assessment system across prefecture-level cities in Sichuan Province confirmed the predictive validity of the evaluation framework. Second, cross-validation between the risk model and coupling model revealed a significant negative correlation, demonstrating that improved coupling coordination degree effectively reduces systemic risks. Finally, verification analysis between regional case growth increments and coupling coordination degree further ensured the robustness of research findings. These validation results provide a solid empirical foundation for subsequent policy recommendations.

Building upon these research findings, this study proposes differentiated governance strategies:

1) Optimization strategies for the Chengdu Plain region

Given Chengdu’s distinctive “low vulnerability-high risk” profile, systemic optimization strategies should be implemented. First, within the Chengdu-Chongqing Economic Circle framework, priority should be given to developing regional growth poles such as Mianyang Science City and the Yibin-Luzhou industrial corridor. This can be achieved through industrial policy guidance and infrastructure interconnectivity to promote polycentric network development, thereby effectively alleviating single-core agglomeration pressures.

Second, urban spatial expansion should incorporate enhanced ecological resilience planning. This includes reserving ecological buffer zones along the outer ring expressway and connecting them through greenway systems to form emergency evacuation networks. Simultaneously, new urban developments should mandatorily reserve convertible emergency land parcels with pre-installed medical equipment interfaces.

Furthermore, structural adjustments to medical resources should be implemented via a “branch hospital system + tiered diagnosis and treatment” model.

These measures will ultimately establish a multidimensional prevention system encompassing “spatial decentralization—ecological buffering—emergency preparedness—medical resource balancing,” systematically mitigating public health risks in high-density urban areas.

2) Balanced development pathway for northeastern sichuan region

To address the concentration of impoverished populations and inadequate infrastructure in northeastern Sichuan, a systematic development strategy should be adopted.

First, priority should be given to cultivating competitive local industries such as specialty agriculture and green processing manufacturing, which can be achieved by establishing return-to-hometown entrepreneurship subsidies and industrial development funds to reverse outmigration trends and boost local employment.

Second, the returning population should be leveraged to drive local fiscal revenue growth, with newly increased public finances being preferentially allocated to upgrading medical facilities and improving transportation infrastructure to enhance public service delivery.

Ultimately, this integrated approach will establish a virtuous development cycle of “industrial revitalization → population agglomeration → fiscal expansion → service improvement,” fundamentally strengthening regional resilience. This cyclical model not only addresses current infrastructure deficiencies but also achieves sustainable development through endogenous growth drivers.

3) Transformation strategies for environmentally stressed cities

For environmentally stressed cities like Yibin and Nanchong, a development path coordinating industrial transformation with environmental governance should be adopted.

First, ecological transformation of key industries should be implemented to reduce pollution intensity through industrial structure optimization.

Second, environmental protection investments should be significantly increased through creating ecological compensation mechanisms, designating health protection buffer zones around major industrial parks, and building a tiered environmental risk prevention system.

Additionally, supporting environmental health monitoring networks should be constructed to enable real-time warnings of pollution sources and health risks.

Through this triple-intervention approach of “industrial upgrading → environmental governance → health protection,” environmentally high-risk cities can transition toward green development models.

4) Optimization and upgrading directions for Panzhihua

As a successfully transitioned resource-based city, Panzhihua has demonstrated outstanding performance in industrial-spatial-ecological coordination, yet requires targeted enhancements in healthcare and social security systems.

First, healthcare system improvements should be prioritized by increasing medical expenditure ratios and optimizing resource allocation.

Second, the current emergency supplies reserves are insufficient, necessitating the establishment of a three-tier allocation system encompassing municipal, county, and mining district levels.

Third, social insurance coverage expansion should be accelerated, particularly for vulnerable groups like miners, through customized insurance schemes and awareness campaigns.

Through this integrated approach of “medical investment → emergency preparedness → insurance coverage,” the city will systematically address existing gaps while reinforcing its sustainable development advantages.

5) Sustainable development pathway for Guangyuan City

As a exemplary model of resilient mountain city development, Guangyuan has achieved remarkable success in spatial-social-environmental coordination, yet requires focused improvements in economic development levels.

First, economic scale expansion should be prioritized by addressing the below-average per capita GDP through targeted growth initiatives.

Second, industrial support strengthening should be implemented by cultivating regionally influential leading industries to address the current lack of driving industries.

Third, fiscal capacity building should be enhanced by improving financial self-sufficiency rates and accelerating infrastructure development to overcome current limitations.

Through this integrated approach of “specialty industry cultivation → fiscal mechanism innovation → infrastructure improvement,” the city will effectively enhance endogenous growth drivers and sustainable development capacity.

## 5 Conclusion

This study innovatively constructs a “hazard-vulnerability” risk coupling model, introducing spatial coupling theory into the field of public health risk management. By employing the entropy method and coupling coordination degree model, it reveals significant spatial heterogeneity characteristics and dynamic interaction mechanisms between infectious disease transmission risks and urban systems, overcoming the limitations of traditional linear risk assessment approaches. Furthermore, the study identifies key factors across different coupling coordination types, providing scientific evidence for formulating differentiated risk prevention strategies and urban resilience solutions.

The results indicate that the coupling coordination degree (CCD) between infectious disease hazards and urban vulnerability in Sichuan Province remains at a relatively low level overall (mean = 0.384). Panzhihua and Guangyuan demonstrate optimal coordination states (with CCD values of 0.649 and 0.655 respectively), while Chengdu exhibits a characteristic “low vulnerability-high hazard” pattern (CCD = 0.031). The region displays a distinct core-periphery spatial differentiation pattern, where cities with imbalanced development are predominantly concentrated in the Chengdu Plain and southern Sichuan regions—a distribution showing significant correlation with the socioeconomic gradients of Sichuan’s four major economic zones. Notably, further typological analysis reveals a prevalent “hazard-dominant lagging” coupling coordination characteristic across the province, which has been corroborated by vulnerability assessments of urban public health systems in relevant planning documents.

Further analysis reveals dual characteristics in the mechanisms influencing urban coupling coordination degree (CCD): on one hand, there exist significant interactions among economic-spatial-social-environmental factors; on the other, these coupling relationships demonstrate pronounced spatial heterogeneity. The study demonstrates that regional coordinated development requires differentiated governance strategies: the Chengdu Plain region should focus on constructing a multidimensional prevention system encompassing “spatial decentralization—ecological buffering—emergency preparedness—medical resource balancing”; northeastern Sichuan needs to establish a virtuous cycle of “industrial revitalization—population agglomeration—fiscal expansion—service improvement”; environmentally stressed cities should prioritize coordinated transformation through “industrial upgrading—environmental governance—health protection”; transition cities like Panzhihua must perfect their social security networks via “medical investment—emergency preparedness—insurance coverage”; while resilient mountainous cities such as Guangyuan should strengthen their development pathway through “specialty industry cultivation—fiscal mechanism innovation—infrastructure improvement.”

This study establishes an analytical framework that provides crucial decision-making support for regional development planning and public health emergency system construction in Sichuan Province. Specifically, the differentiated intervention strategies proposed for regions with distinct characteristics can guide the optimization of public health resource allocation and the implementation of urban resilience-building projects. The research outcomes not only offer valuable references for infectious disease prevention and control in rapidly urbanizing areas, but also provide theoretical foundations for achieving healthy city initiatives and sustainable development goals.

It should be noted that this study still has several aspects requiring further development: First, the factor analysis in urban vulnerability assessment needs strengthening, and future research could introduce more comprehensive spatial variables and governance effect variables to improve the evaluation system. Second, while the entropy method can objectively reflect data characteristics, its results may be influenced by data distribution—subsequent studies could incorporate other weighting determination methods like the Analytic Hierarchy Process for verification. Finally, the applicability of research conclusions drawn from western provinces to eastern developed regions requires validation. Future studies should expand the research scope and conduct comparative analyses of regional characteristics at different development stages to enhance the generalizability of findings.

## Data Availability

The data analyzed in this study is subject to the following licenses/restrictions: the data presented in this study are available on request from the corresponding author. The data are not publicly available due to privacy. Requests to access these datasets should be directed to the corresponding author at qiujian@home.swjtu.edu.cn.

## Footnotes

^1^ https://wsjkw.sc.gov.cn/

^2^ https://lbs.amap.com

^3^ https://www.tianditu.gov.cn/
